# Automated estimation of computed tomography-derived left ventricular mass using sex-specific 12-lead ECG-based temporal convolutional network

**DOI:** 10.1093/ehjdh/ztaf122

**Published:** 2025-10-22

**Authors:** Heng-Yu Pan, Benny Wei-Yun Hsu, Chun-Ti Chou, Yuan-Yuan Hsu, Chih-Kuo Lee, Wen-Jeng Lee, Tai-Ming Ko, Vincent S Tseng, Tzung-Dau Wang

**Affiliations:** Division of Cardiology, Department of Internal Medicine, National Taiwan University Hospital Hsin-Chu Branch, Hsin-Chu City, Taiwan; Department of Biological Science and Technology, National Yang Ming Chiao Tung University, Hsin-Chu City, Taiwan; Department of Computer Science, National Yang Ming Chiao Tung University, No. 1001, Daxue Rd, E District, Hsin-Chu City, 300093, Taiwan; Department of Computer Science, National Yang Ming Chiao Tung University, No. 1001, Daxue Rd, E District, Hsin-Chu City, 300093, Taiwan; Department of Computer Science, National Yang Ming Chiao Tung University, No. 1001, Daxue Rd, E District, Hsin-Chu City, 300093, Taiwan; Division of Cardiology, Department of Internal Medicine, National Taiwan University Hospital Hsin-Chu Branch, Hsin-Chu City, Taiwan; Department of Medical Imaging, National Taiwan University Hospital, Taipei City, Taiwan; Department of Biological Science and Technology, National Yang Ming Chiao Tung University, Hsin-Chu City, Taiwan; Department of Computer Science, National Yang Ming Chiao Tung University, No. 1001, Daxue Rd, E District, Hsin-Chu City, 300093, Taiwan; Cardiovascular Center and Division of Cardiology, Department of Internal Medicine, National Taiwan University Hospital, No. 7, Zhong-Shan South Road, Taipei City 100225, Taiwan

**Keywords:** Left ventricular mass, Temporal convolutional network, Electrocardiogram, Deep learning, Sex-specific models, Cardiac imaging

## Abstract

**Aims:**

To propose a novel deep learning-based method, the eLVMass-Net, for the estimation of left ventricular mass (LVM) based on 12-lead electrocardiograms (ECGs).

**Methods and results:**

We developed a deep learning model for LVM estimation using raw ECG signals, demographic data, and ECG parameters as input by using TW-CVAI dataset (*n* = 1459). Synchronized single-heartbeat waveforms were processed using a temporal convolutional network (TCN). Ground-truth LVM values were obtained from coronary computed tomography angiography. We performed external validation on an independent NTUH dataset (*n* = 2579). To account for sex-specific differences in left ventricular remodelling and body habitus, we further developed separate models for males and females. We compared the performance of the eLVMass-Net, with two state-of-the-art (SOTA) models.

Non-sex-specific eLVMass-Net achieved a mean absolute error (MAE) of 14.3 ± 0.7 g and a mean absolute percentage error (MAPE) of 12.9 ± 1.1% between predicted and ground-truth LVM values under five-fold cross-validation. The eLVMass-Net outperformed two SOTA models in terms of both LVM estimation and left ventricular hypertrophy (LVH) classification. Sex-specific design was superior in LVH classification based on estimated LVM (*c*-statistic: 0.77 ± 0.05 for male model; 0.75 ± 0.05 for female model; 0.70 ± 0.02 for non-sex-specific model; *P*  *<* 0.01 between both sex-specific models vs. non-sex-specific model). The saliency maps revealed gender-specific differences in how the model weighted ST-T segment features for LVM prediction.

**Conclusion:**

The proposed eLVMass-Net outperformed previously published approaches by ECG pre-processing with synchronized single heartbeat extraction and TCN as ECG encoder. Additionally, the development of sex-specific models proved to be a rational approach.

## Graphical Abstract

**Graphical Abstract:**
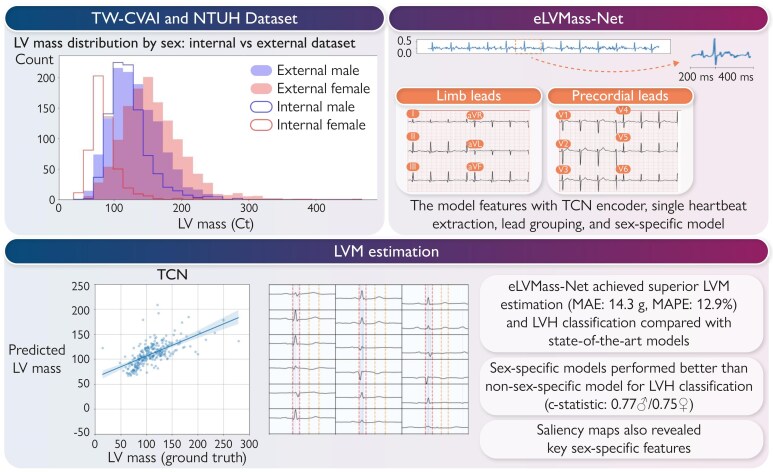

## Introduction

Left ventricular hypertrophy (LVH) is defined by an increased left ventricular mass (LVM), usually secondary to conditions with higher left ventricular afterload, like long-standing hypertension and aortic stenosis. LVH is a dynamic pathophysiological phenomenon with concomitant changes in cardiomyocytes and interstitial fibrosis.^1,2^ Further progressions in LVH are associated with diastolic dysfunction, arrhythmia as well as cardiac death.^3–5^ Traditionally, the diagnosis of LVH relies on various rule-based criteria, mainly focusing on QRS voltage presented on individual electrocardiogram (ECG), which is a low cost and convenient test.^6^ Nonetheless, most of these criteria often fall short of sensitivity.^7–9^ It is not surprising considering that commonly used criteria such as Sokolow–Lyon or Cornell voltage criteria are negatively correlated to the degree of left ventricular fibrosis.^10^ It is assumed that comprehensive ECG features being considered in order to encompass the electrophysiological traits.^11^ Unlike ECG, both cardiac magnetic resonance (MR) and computed tomography (CT) are able to provide accurate measurement of LVM.^12,13^ However, these imaging modalities are either time-consuming or associated with radiation and contrast exposure. Also, such information provides anatomical rather than electrophysiological features.

Nowadays, machine learning models are used for ECG feature extraction in the aim of either LVM estimation or LVH classification.^14–16^ Studies reported an overall sensitivity for prediction of LVH ranges from 0.34 to 0.97, the specificity from 0.57 to 0.96, and overall *c*-statistic from 0.62 to 0.92.^14,17–27^ These studies used at least 10 s of 12-lead ECG waveform data for feature extraction by exploiting ensembled supervised learning, convolutional neural network (CNN), or transformer-based ECG encoders. Half incorporated demographic data as distinct input features, and three included ECG parameters additionally. Two research groups used LVM values and LVH inferred from cardiac MR images as ground truth, whereas others relied on echocardiography-based LVH diagnosis. It is debatable considering that echocardiography tends to overestimate LVM in comparison with either MR or CT despite its better accessibility.^28,29^

In this study, we developed a deep-learning model for LVM estimation based on the Taiwan CardioVascular Artificial Intelligence (TW-CVAI) dataset. We accessed demographic data, 12-lead ECG, and CT-derived LVM values, then utilized these data to construct an LVM estimation model. Meanwhile, we conducted a series of experiments to analyse the impact of various ECG pre-processing methods, lead grouping strategies, combination of input features, and sex-specific settings in order to improve model performance. Our proposed models were compared with two state-of-the-art (SOTA) models.

## Methods

### Data acquisition

**Figure 1:**
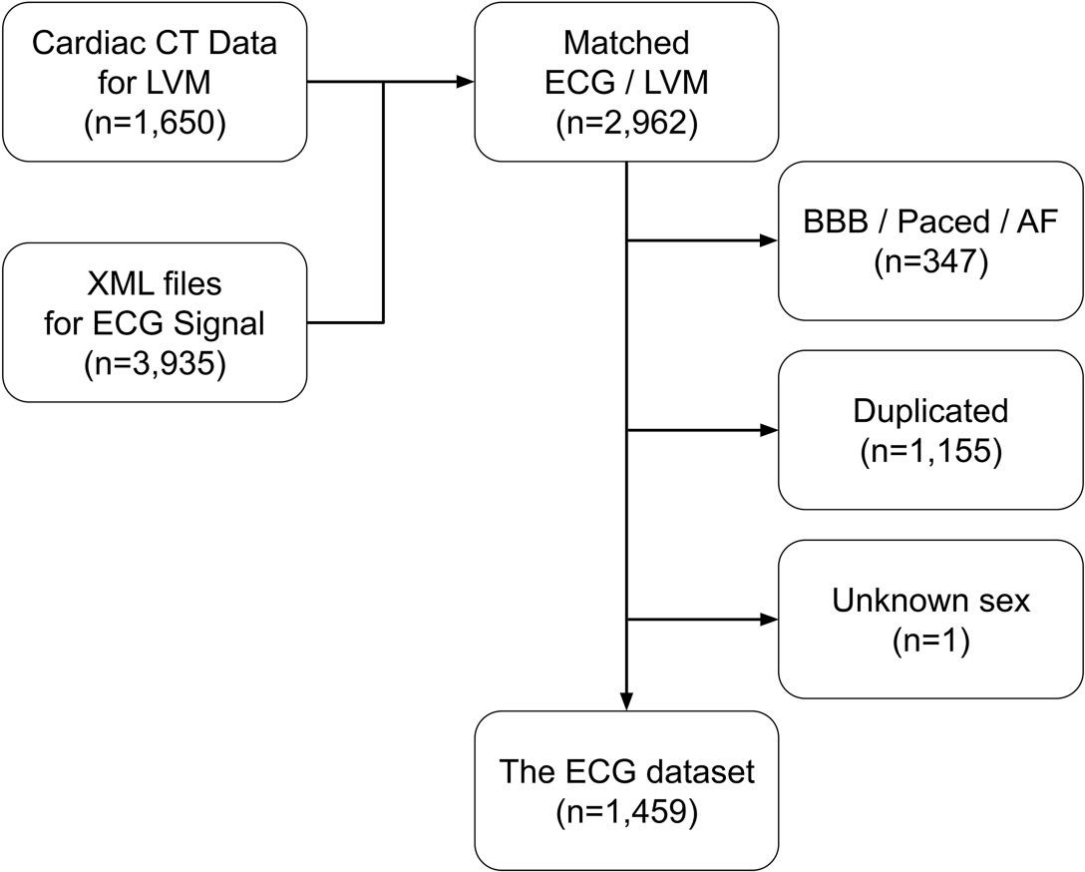
Overview of dataset collection. The computed tomography data and corresponding XML files were collected independently. A matching process was carried out based on the requirement that both measurements be taken within 6 months. Patients with bundle branch block, paced rhythm, and atrial fibrillation were excluded due to distorted electrocardiogram waveforms. Electrocardiogram recordings that did not have a one-to-one paired left ventricular mass measurement were also excluded. A total of 1459 valid data points were included in this study. AF, atrial fibrillation; BBB, bundle branch block.

The TW-CVAI dataset consists of demographic data, 12-lead ECGs, and coronary CT angiography (CCTA) images. Pertinent demographic data such as age, sex, height, and weight are included. ECGs were recorded at a sampling rate of 500 Hz for 10 s and downloaded in XML format. The XML files also contain automatically generated ECG parameters, such as QRS axis and duration. *Figure 1* shows the overall data collection and cleansing process. The LVM values were first obtained from CCTA (*n* = 1650), and all ECGs pertained to the same patient were included (*n* = 3935). We identified 2962 ECGs performed within 6 months of CCTA, while 1155 ECGs were excluded for redundancy (i.e. multiple ECGs matched with one specific LVM value). Patients with bundle branch block, paced rhythm, atrial fibrillation or unknown sex were excluded from the research (*n* = 348). Eventually, 1459 patients with accessible demographic, ECG, and CCTA were finally included. The use of TW-CVAI dataset was approved by National Taiwan University Hospital Research Ethics Committee (No. 202012128RINA).

The LV myocardium volume was inferred from auto-segmentation of left ventricular wall from CCTA images, by using the Intellispace Portal Software (Philips Healthcare, The Netherlands). LVM was further acquired after multiplying LV myocardium volume with the density of 1.05 g/mL. The results of left ventricle segmentation were verified by a senior radiologist who is specialized in CCTA images and with more than 20 years of experience.

The dataset was divided into non-overlapping subsets for cross-validation. Five-fold cross-validation was employed to prevent sampling bias. The five-folds were formed by sorting the dataset based on the target LVM values before selecting indices. The dataset was then split systematically by picking every one in five samples for each fold to ensure an even distribution of LVM. In each iteration, a single fold was reserved as the test set (*n* = 292 ± 1), while a subset of the training set (*n* = 1051 ± 1) was further selected as the validation set (*n* = 116 ± 1) to assess model performance. The selection of the optimal model was based on the performance observed during the validation stage. The final evaluation of the model was obtained by averaging the test results across all folds. For sex-specific models, the original data splitting policy was followed, and the samples of the target gender were extracted to form the sub-datasets for training and evaluation.

External validation was performed on a retrospective cohort with patients having attainable ECG and echocardiography-derived LVM values within 6-month period in between September and December 2023. These patients received outpatient care at National Taiwan University Hospital (NTUH) (*n* = 2579). The dataset was preliminarily extracted and curated by the Integrative Medical Data Center, National Taiwan University Hospital (NTUH-iMD). Considering that echo tends to overestimate LVM, we recalibrated echo-derived LVM to CT-/MR-derived LVM using a previously published formula as final ground-truth.^30^ The accuracy of eLVMass-Net was indicated by mean absolute error (MAE) and mean absolute percentage error (MAPE) between predicted and ground-truth LVM. Same exclusion criteria for ECG applied for this NTUH dataset. The use of NTUH dataset was approved by National Taiwan University Hospital Research Ethics Committee (No. 202407110RIND).

### Data pre-processing

The ECG signals underwent a band-pass filter to eliminate high-frequency noise and baseline wandering. R peaks were detected and heartbeats were segmented within a fixed time interval around R peak to include P, QRS, and T wave. For demographic data and ECG parameters, missing data were addressed through an imputation method as numerical data were imputed with the median.

### ECG pre-processing

Relevant SOTA models for LVM estimation required input of at least 10-s ECG waveform data. The waveform data either went through ECG segmentation and vector transformation or were passed directly to deep neural networks for feature extraction.^14,21^ In addition to whole length, we explored the use of synchronized random length crop or single heartbeat extraction to guide the model to learn ECG features. The random length crop approach involves randomly selecting an ECG segment of varying time lengths which are synchronized over 12 leads. It is a common method for data augmentation, which could enhance diversity and reduce susceptibility to noises. On the other hand, the single heartbeat extraction approach involves segmenting the middle single heartbeat synchronized over 12 leads. A single heartbeat segment could require less computational resources and be less susceptible to interference from irrelevant noise compared with longer segments.^31^

We further investigated the use of lead grouping to improve the prediction accuracy of ECG signals. Lead grouping is mainly based on domain knowledge that certain leads belong to same electrical vector projection plane or anatomical location. The spatial relationship provided by lead grouping improved the diagnostic performance on multilabel classification tasks involving structural heart disease such as myocardial infarction or left ventricular hypertrophy.^32^ The strategy has never been used for any of the relevant methods for LVM estimation. We applied lead grouping on the 12-lead ECG signals either based on electrical signal vector plane orientation or anatomical location. For electrical vector orientation, 12-lead ECG signals are grouped into two separate groups based on horizontal and frontal planes (i.e. precordial and limb leads). For anatomical location, we formed four groups based on the distribution of coronary artery branches within the heart. Leads V1–V4 were grouped as leads related to the left anterior descending artery. Leads I, aVL, V5, and V6 were grouped as leads related to the left circumflex artery, whereas leads II, III, and aVF and lead aVR were related to the right coronary artery and the left main coronary artery, respectively.

### eLVMass-net

**Figure 2:**
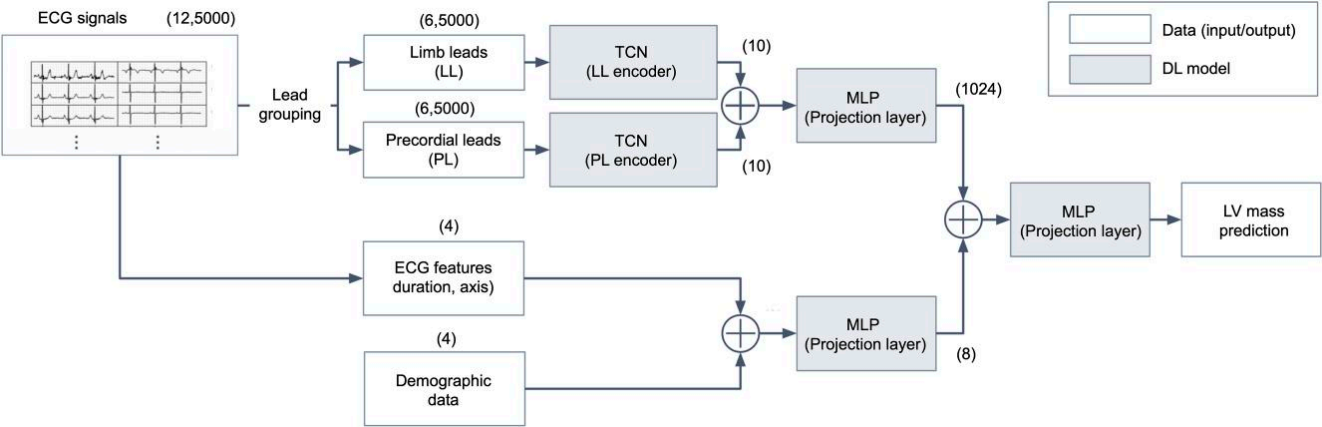
Overview of the proposed left ventricular mass estimation framework. The proposed left ventricular mass estimation model consists of separate encoders for the limb leads and chest (precordial) leads of the 12-lead electrocardiogram , followed by a multilayer perception layer. Scalar features such as demographic data, heart axis, and QRS duration are passed through their own multilayer perceptron layer. The encoded electrocardiogram features and scalar features are then concatenated and fed into the prediction layer to estimate the left ventricular mass. DL, deep learning; LV, left ventricular; TCN, temporal convoluted network.

We developed a novel deep learning-based method, the eLVMass-Net, which can accurately estimate LVM values using ECG signals, demographic data and ECG parameters. The overview of the proposed framework is depicted in *Figure 2*. There are ECG feature extractors (i.e. the encoders for input data embedding) followed by a multilayer perceptron (MLP) layer. To obtain ECG features, we intended to use networks with more flexibility in temporal resolution.^33–35^ We took advantage of the temporal convolutional networks (TCN) to encode ECG signals. TCN is a type of deep neural network that is ideal for processing sequential data, such as time-series signals. TCNs use dilated convolutions, enabling the network to capture long-term dependencies in the input sequence while maintaining a compact architecture. The number of ECG encoders utilized in the model is dependent on the number of leads. The encoded ECG features from each ECG encoder were concatenated, and a projection layer was utilized to aggregate these features. The scalar features such as demographic data and ECG parameters were passed through their own MLP layers. These two feature vectors were subsequently concatenated with the ECG waveform vectors and fed into an MLP regressor to estimate the LVM value. Furthermore, we developed sex-specific models in consideration of significant gender differences in LVH and body habitus.^36^ Sex-specific models are rarely reported in previous relevant literatures, particularly the SOTA methods.^26^ The hyperparameters used in model training are shown in Supplementary material online, *Supplementary Methods*. Throughout the process of model training, the MAE was employed as the loss function.

### Combinations of input data

We designed three input combinations in our study to better understand how different combinations contribute to the overall model. The first input set included only raw ECG waveform signals, which is a commonly used and simple setting that can be applied to any 12-lead ECG datasets. This setting served as a baseline for comparison with more complex input sets. Demographic data was added as the second input set, given this information providing a general description of physiological conditions that may affect electric conductance from the heart and various heart functions. Demographic data are widely available in most clinical fields, making it a useful addition to the input set. Lastly, we included ECG parameters as the third input set upon previous combinations. Cai *et al*. and Ryu *et al*. used a wide range of ECG parameters in their studies.^18,26^ We opted to exclude heart rate, QT interval and rhythm diagnosis as those parameters require information from multiple heartbeats, with which the information could be limited from shortened ECG segments. R/S amplitudes of selective ECG leads were not measured or served as input parameters, as they are not routinely available in automatic ECG interpretation. Our final combination of ECG parameters was QRS duration, *P* axis, *R* axis and *T* axis. As a sensitivity analysis, we also experimented a fusion model based on purely ECG signals and parameters. Before the experiment, we explored the relationship between demographic data/ECG parameters and LVM values by using both Pearson and Spearman correlations.

### Comparison with SOTA methods

We compared the performance of eLVMass-Net with two relevant models, ECG-AI and LVM-AI, based on their contributions to LVM estimation and LVH prediction.

LVM-AI utilizes 10-s, 12-lead ECG signals, which are processed through a 10-layer CNN with age, sex, and body mass index (BMI) as additional inputs. These ECG signals are used without further pre-processing, and the ground truth for LVM estimation is derived from cardiac MR.

In contrast, ECG-AI processes 10-s ECG signals using a pre-trained CNN and hidden Markov models (HMMs) for feature extraction before passing them to a regression-tree-based machine learning model. Unlike LVM-AI, ECG-AI does not require additional demographic inputs. This model estimates multiple left ventricular attributes, including LVM, and its predictions are compared with echocardiography-derived LVM.

To ensure a fair comparison, we accessed the source codes and model architecture from the original publications. We retrained the models using our internal dataset rather than adopting pre-trained weights. We further adapted these SOTA models to our data with consistent pre-processing across methods. This ensured a fair comparison under the same data distribution and evaluation criteria.

### Statistical analysis

To evaluate the performance of the predicting model, MAE and MAPE were utilized. These widely accepted metrics are commonly employed in regression analysis to gauge the accuracy of predicted values in comparison to actual values. MAPE calculates the absolute percentage difference between predicted and actual values and averages them over the dataset, while MAE measures the average absolute difference between the predicted and actual values. The *P*-values are calculated across folds, meaning they are derived from the aggregated results of all cross-validation folds rather than individual subjects.

To compare the diagnostic performance of our model, we further calculated accuracy, sensitivity, positive predictive value, specificity, F1 score, and *c*-statistic with the reference of LVH inferred by CCTA. LVH is defined as an indexed LVM > 72 g/m^2^ for men or >55 g/m^2^ for women.^37,38^ The cut-off values were used considering that both MR- and CT-derived indexed LVM are in good agreement.^39^

We also used the saliency maps from the proposed framework to assess the importance of different ECG segments. The saliency maps were obtained using gradient-based saliency visualization by computing the gradients of the model’s output with respect to the input ECG signals. Backward pass was performed on the model’s prediction to obtain gradients that indicate feature importance. The absolute values of these gradients were then extracted and visualized as heatmaps overlaid on the original ECG signals, highlighting the most influential regions for the model's prediction. For further illustration, the ECG signal was divided into non-overlapping segments, including the PR interval, QRS interval, ST-T segment (including ST segment and T-wave), and TP interval. Subsequently, the significance of each segment was determined by calculating the summation of their respective significance scores, which represent the overall segment significance.

## Results

### Patient characteristics

**Figure 3:**
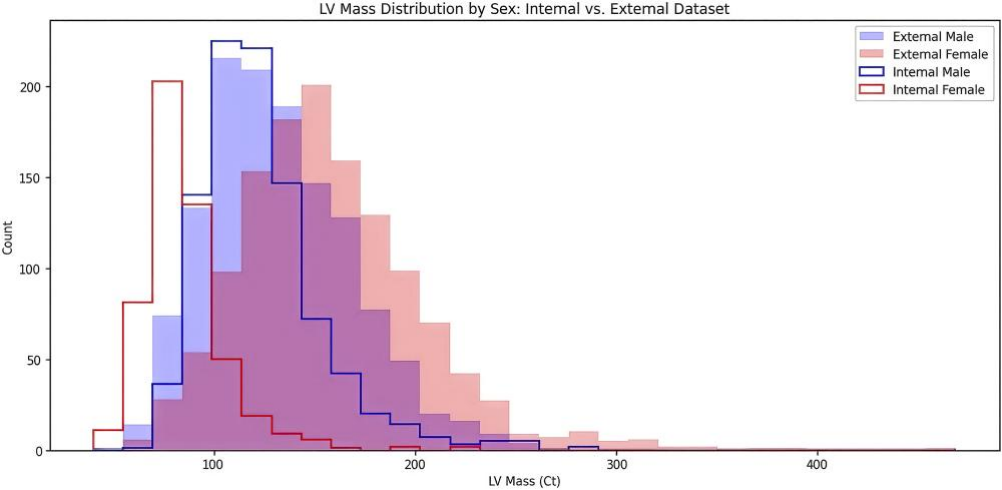
Sex-specific left ventricular mass distribution across datasets.

The sex-specific patient characteristics of TW-CVAI and NTUH datasets are summarized in Supplementary material online, *Table S1*. Compared with female patients, male patients generally had wider QRS segments and more leftward axis. Generally speaking, there are moderate to strong positive correlations between LVM and features including height, weight and QRS duration, and weak negative correlation between LVM and R axis (see Supplementary material online, *Figure S1*). *Figure 3* presents the sex-specific distribution of LVM across the TW-CVAI and NTUH datasets (used for external validation). Notably, the external dataset shows a clearly right-skewed distribution of LVM among females.

### Effect of ECG grouping and pre-processing methods

For the assessment of ECG pre-processing, data from both sexes were utilized as the training data. The results of using different ECG pre-processing techniques are shown in *Table 1(a)*. We found that the synchronized single heartbeat extraction can significantly improve the performance of the deep learning model in predicting LVM. On the contrary, data augmentation by random length crop did not improve model performance compared with full-length input. The experiments on lead grouping suggested that grouping based on electrical vector orientation had an insignificant improvement on model performance than non-grouping method [*Table 1(b)*].

**Table 1:** Left ventricular mass prediction performance with (a) ECG pre-processing; (b) lead grouping; and (c) multimodal input combinations

	MAE (g)	MAPE (%)	*P* value
**(a) ECG pre-processing**			
Full length	14.9 (±0.7)	13.5 (±0.8)	<0.001
Random length crop	15.1 (±0.5)	13.6 (±1.0)	<0.001
Single heartbeat (eLVMass-Net)	14.3 (±0.7)	12.9 (±1.1)	—
**(b) Lead grouping**			
Non-grouped	14.7 (±0.6)	13.2 (±1.0)	0.34
Anatomical location	14.8 (±0.7)	13.4 (±1.2)	0.31
Electrical orientation (eLVMass-Net)	14.3 (±0.7)	12.9 (±1.1)	—
**(c) Multimodal combinations**			
ECG	19.2 (±0.9)	17.4 (±1.4)	<0.001
ECG + parameters	19.1 (±1.1)	17.2 (±1.2)	<0.001
ECG + demographics	14.6 (±0.5)	13.0 (±1.0)	0.85
ECG + demographics + parameters (eLVMass-Net)	14.3 (±0.7)	12.9 (±1.1)	—

The model performance under different ECG pre-processing, lead grouping, and input combinations were compared with eLVMass-Net. *P*-values were calculated by comparing these methods to the outcomes of eLVMass-Net. Both MAE and MAPE were calculated using the validation samples from five-fold cross-validation (*n* = 292 ± 1) and presented in the form of mean (±standard deviation). ECG, electrocardiogram; MAE, mean absolute error; MAPE, mean absolute percentage error.

#### Sensitivity analyses

The results of different input combinations are shown in *Table 1(c)*. Demographic data played a crucial role in predicting LVM using our model. The inclusion of demographic data significantly improved the prediction performance compared to using only raw ECG signals by 25.1% (*P*  *<* 0*.001*). Further addition of the ECG parameters provided an insignificant performance improvement (by an absolute difference of 0.7%, *P*  *=* 0*.82*). The fusion model using only ECG signals and parameters performed worse than the complete model.

### Model validation using eLVMass-net and SOTA methods

We conducted performance comparisons using both internal (TW-CVAI) and external (NTUH) datasets. All available features (ECG signals, demographic data, ECG parameters) are used, showcasing the advantages of our approach while improving the SOTA methods as well (see *Table 2*).

**Table 2:** The performance of the eLVMass-Net and the SOTA models

	MAE (g)		*P*-Value	MAPE (%)		*P*-value
Model name	Original setting	All features		Original setting	All features	
ECG-AI	29.6 (±0.9)	21.3 (±0.4)	<0.01	26.9% (±1.2%)	19.1% (±0.6%)	<0.01
LVM-AI	19.6 (±0.9)	19.5 (±0.8)	0.04	17.5% (±1.3%)	17.6% (±0.8%)	0.07
eLVMass-Net	—	14.3 (±0.7)	—	—	12.9% (±1.1%)	—

Both MAE and MAPE were calculated using the validation samples from five-fold cross-validation (*n* = 292 ± 1) and presented in the form of mean (±SD). The *P*-values were computed by comparing these methods to the results of eLVMass-Net. Both MAE and MAPE are presented in the form of mean (±SD).

For TW-CVAI test set, the eLVMass-Net has achieved the lowest MAE of 14.3 g and MAPE of 12.9% among all the methods (MAE and MAPE for ECG-AI, 21.3 g and 19.1%; for LVM-AI, 19.5 g and 17.6%). Performance metrics for LVH classification were generally better for eLVMass-Net compared with the other two SOTA methods (*c*-statistic = 0.70 ± 0.02, accuracy = 0.77 ± 0.33, sensitivity = 0.47 ± 0.04, specificity = 0.89 ± 0.03, PPV = 0.65 ± 0.06, F1 score = 0.54 ± 0.04) (see Supplementary material online, *Table S2*). For external validation on NTUH dataset, the eLVMass-Net achieved an MAE of 29.2 g and MAPE of 20.3%. The ECG-AI model trained on the TW-CVAI dataset had inferior performance with an MAE of 42.1 g and MAPE of 44.3% (*P*  *=* 0.03). In contrast, the LVM-AI model trained on the same internal dataset demonstrated poor adaptability to the external dataset, yielding a high MAE of 155.6 g and exceedingly high MAPE (*P*  *<* 0.001) (see Supplementary material online, *Table S3*).

### Sex-specific analysis

When compared to the non-sex-specific eLVMass-Net model, male model demonstrated a relative improvement of 2.7% in terms of MAPE (*P*  *=* 0.30), and female model had a relative improvement of 3.0% (*P*  *=* 0.10) (see Supplementary material online, *Table S4*). LVH discrimination was better for both sex-specific eLVMass-Net models (*c*-statistic: 0.77 ± 0.05 for male model; 0.75 ± 0.05 for female model; 0.70 ± 0.02 for non-sex-specific eLVMass-Net model; *P*  *=* 0.008 for male model vs. non-sex-specific model and *P*  *<* 0.001 for female model v. non-sex-specific model) (see Supplementary material online, *Table S5*).

### Saliency maps and gender differences

Samples (*n* = 5 for each) of low, medium, and high LVM values were selected. The mid part of the T wave in precordial leads and the QRS segment in limb leads are highlighted as important features with increasing LVM. *Table 3* presents a non-sex-specific summary of the segment-wise significance of the input ECG. It shows that the importance is mainly concentrated in segments of QRS interval and ST-T segment. Furthermore, the importance of precordial leads decreased as the LVM value increased (62.2% for the low LVM group and 40.0% for the high LVM group).

**Table 3:** ECG-segment-wise importance (%) for each segment from saliency maps from both non-sex-specific model and sex-specific models

	Non-sex-specific						Male						Female					
LVM	Low		Middle		High		Low		Middle		High		Low		Middle		High	
	LL	PL	LL	PL	LL	PL	LL	PL	LL	PL	LL	PL	LL	PL	LL	PL	LL	PL
PR interval	3.5	0.5	3.1	0.0	3.9	0.0	1.0	0.0	0.5	0.0	0.2	0.0	0.0	0.0	4.4	0.0	1.5	0.4
QRS interval	34.3	42.9	38.0	41.8	53.3	17.5	34.8	53.7	39.0	22.8	49.2	9.2	36.5	49.2	34.5	30.8	54.7	26.2
ST-T segment	0.0	18.8	0.5	16.6	2.8	22.5	0.0	10.5	0.0	37.7	7.0	34.4	0.0	14.3	0.0	30.3	0.0	17.2
TP interval	0.0	0.0	0.0	0.0	0.0	0.0	0.0	0.0	0.0	0.0	0.0	0.0	0.0	0.0	0.0	0.0	0.0	0.0
Total	37.8	62.2	41.6	58.4	60.0	40.0	35.8	64.2	39.5	60.5	56.4	43.6	36.5	63.5	38.9	61.1	56.2	43.8

The percentages were the averages from five samples.

LVM, left ventricular mass; LL, limb leads; PL: precordial leads.

**Figure 4:**
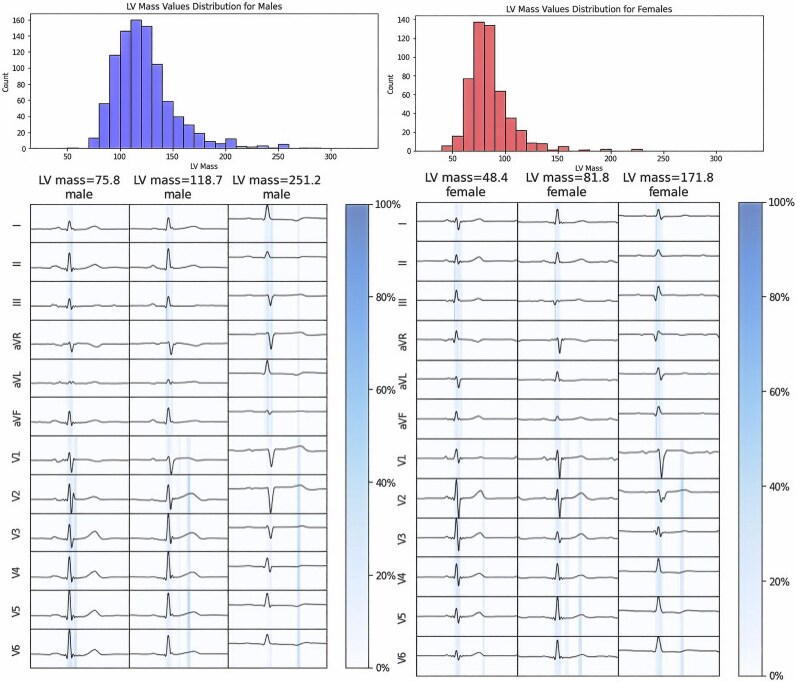
Saliency maps of the eLVMass-Net. Three samples were selected to represent low, middle, and high left ventricular mass values for both males and females. LV, left ventricular.

The segment-wise importance for sex-specific model is also shown in *Table 3*. The saliency maps for sex-specific model are illustrated in *Figure 4*. Notably, the importance of precordial ST-T segment experiences a proportional augmentation for males with increasing LVM values (10.5% for low LVM to 34.4% for high LVM). This trend has not been observed in females (14.3% in low LVM to 17.2% in high LVM). Conversely, both males and females presented with increasing importance of limb QRS segment with increasing LVM (34.8% in low LVM to 49.2% in high LVM for males; 36.5% in low LVM to 54.7% in high LVM for females).

## Discussion

The eLVMass-Net was trained on CT-derived LVM values, 12-lead ECG, and demographic data of around 1500 individual patients. The model's ECG encoder is built on a TCN, specifically designed to capture time-dependent features, unlike conventional 1D CNNs used in previous studies. In addition, our study showed that synchronized single heartbeat extraction is better than whole-length input for LVM estimation. Demographic-inclusive and sex-specific design result in even better accuracy for diagnosing LVH.

Our proposed method, eLVMass-Net, is effective in predicting LVM values with use of TCN encoders and unique ECG pre-processing methods. In the case of the LVM-AI model, the observation of overfitting during training suggests that the model may be too complex or not regularized enough for the size of the dataset used in training. It means that the model has learned to fit the training data very well but needs to generalize better to new data. On the contrary, eLVMass-Net employed TCN to effectively capture time-dependent ECG features. Sensitivity analyses indicated that incorporating QRS axis or duration did not enhance performance, suggesting that the TCN-based ECG encoder sufficiently extracts the necessary temporal information. Compared with the performance between eLVMass-Net and LVM-AI, ours improved by 27% for MAE and MAPE. ECG segmentation labels are necessary for the ECG-AI training pipeline, which is not available in the original XML files. When applying ECG-AI on other datasets, additional effort is needed to label ECG segments. The LVM estimation task will be trained on separate datasets. The experimental results suggest that using a separate dataset for training the ECG segmentation model may have contributed to the low performance of the ECG-AI model on our dataset. It may be due to differences in data distribution, recording devices, or pre-processing steps between the two datasets. Therefore, our proposed method shows a relative improvement of 33% for MAE and MAPE compared to ECG-AI. Sex-specific eLVMass-Net provides better LVH discrimination based on LVM estimation, compared with its counterparts.

The results of this study are to be further interpreted in the clinical context. First, our proposed model focuses more on the QRS interval of limb leads and ST-T segments in precordial leads with increasing LVM. It is proposed that a hypertrophied heart grows disproportionately toward the inferior, leftward, and posterior axes.^40^ Also, T-wave abnormalities may reflect the severity of left ventricular hypertrophy. Respectively integrating both precordial- and limb-lead features by individual encoders may further increase the diagnostic accuracy.^41,42^ Second, previous studies indicated that sex difference exists in QRS duration and voltage regardless of baseline body size or left ventricular mass.^16,43^ Even with similar comorbidities or disease severity, there are significant differences in terms of LVM and extent of myocardial fibrosis between sexes.^44,45^ The sex-specific model revealed better LVH discrimination power compared with the non-sex-specific model. There were distinct differences in segment-wise importance associated with increasing LVM between men and women. Likewise, it was demonstrated that the presentation of either T wave inversions in men or increased precordial voltage in women is associated with heart failure hospitalization.^46^ It is possible that currently developed deep learning algorithms are able to detect important sex-specific pathophysiological differences.^47^

Performance metrics from external validation was less optimal. It is conceivable if we consider the more right-skewed distribution of LVM, derived from echocardiography, in the external NTUH dataset. eLVMass-Net may need dedicated recalibration before being applied to a diverse patient geographic population. Future research should focus on obtaining and incorporating external validation data from diverse geographic regions to further enhance the reliability and generalizability of our approach.

## Conclusions

Accurate assessment of LVM is crucial in diagnosing and managing cardiovascular diseases. We proposed eLVMass-Net as a novel approach that includes TCN encoder and synchronized single heartbeat input for LVM estimation. For gender disparities, the sex-specific model is able to discriminate important ECG features associated with different LVM.

## Lead author biography


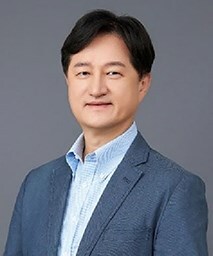


Vincent S. Tseng, Ph.D., Chair Professor at NYCU, Taiwan, specializes in AI, big data, and biomedical informatics. With 400+ publications, 15 patents, and 16 000+ citations, he is an IEEE Fellow and ACM Distinguished Member. Recognized with awards like the PAKDD Distinguished Service Award (2024) and Pan Wen Yuan Foundation Outstanding Research Award (2023), he has advanced AI and data science in academia and industry.


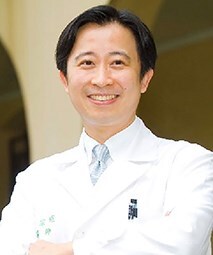


Tzung-Dau Wang, MD, PhD, FESC, is a leading interventional cardiologist and professor of medicine at National Taiwan University Hospital’s Cardiovascular Center. Recognized with the Nong Ding Award (lifetime achievement award) of Taiwan Society of Cardiology (2024), the Outstanding Research Award by National Taiwan University Hospital (2024), the World’s Top 2% Most-Cited Scientist (Stanford/Elsevier) and a Fellow of the European Society of Cardiology, Dr. Wang is dedicated to advancing innovative approaches to hypertension, digital medicine/AI and interventional treatment for hypertension, erectile dysfunction and pelvic arterial diseases.

## Supplementary Material

ztaf122_Supplementary_Data

## Data Availability

The data that support the findings of this study are not publicly available due to privacy and ethical restrictions. Participants did not consent for their data to be shared publicly. As a result, the datasets generated and analysed during the current study are not available.
