# Supplementary material for: Automated estimation of computed tomography-derived left ventricular mass using sex-specific 12-lead ECG-based temporal convolutional network
Source: Eur Heart J Digit Health. 2025 Oct 22;7(1):ztaf122. doi: 10.1093/ehjdh/ztaf122 (PMC12821057; doi:10.1093/ehjdh/ztaf122)
Supplement: ztaf122_Supplementary_Data [file ztaf122_supplementary_data.docx]

**Supplementary method:**

The Adam optimizer, with a learning rate of 0.001, was utilized for model optimization. And with a maximum of 100 epochs, the model with the lowest validation loss was selected for testing to avoid overfitting.


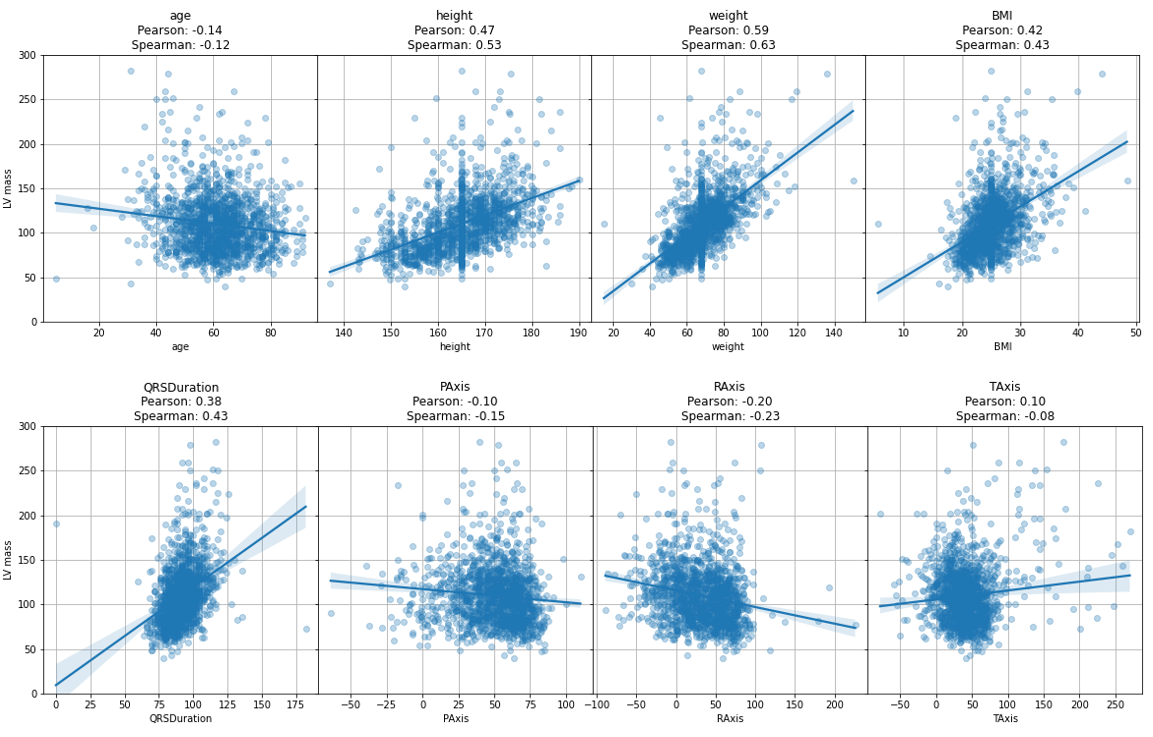

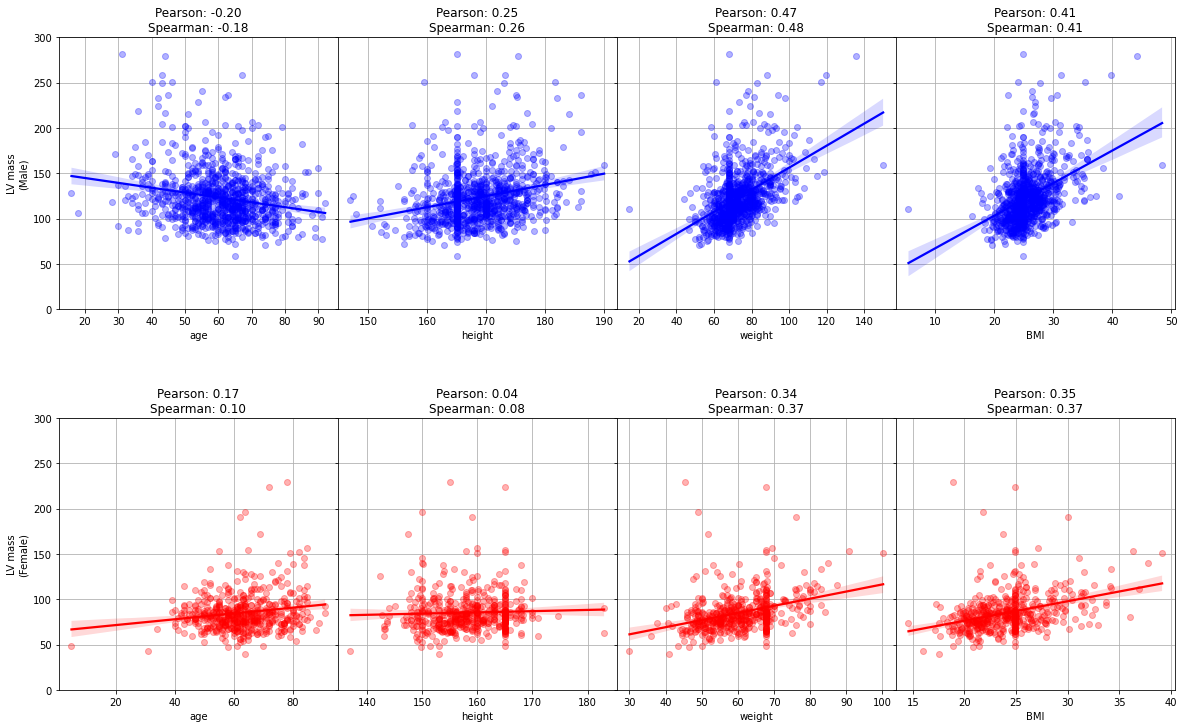

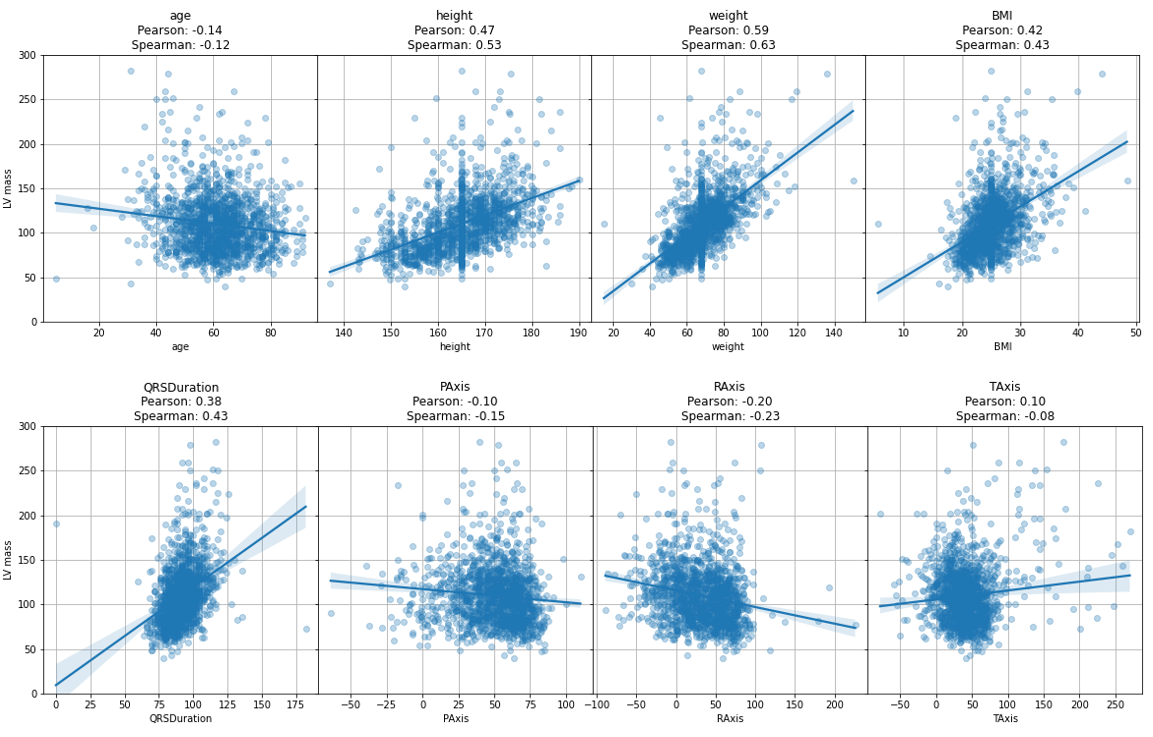

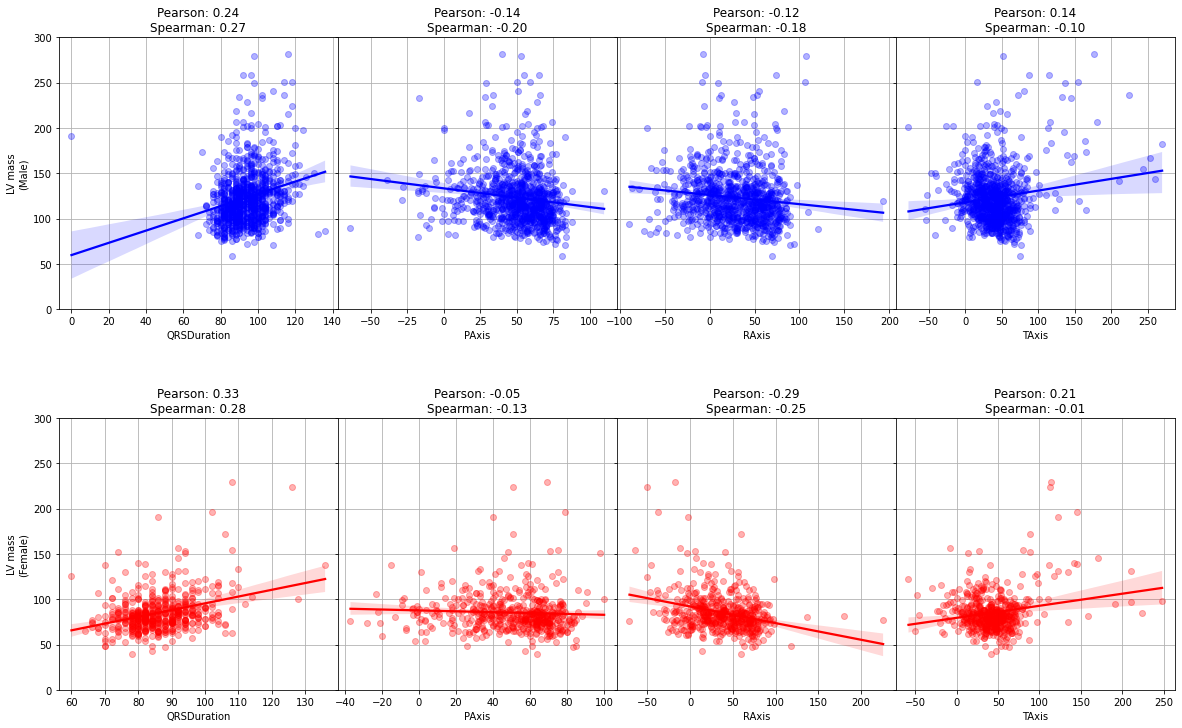


Supplementary Figure 1. Correlation between LVM values and the features, including age, height, weight, BMI, QRS duration, P Axis, R axis, and T axis.

|  | **TW-CVAI (internal)** | | **NTUH (external)** | |
| --- | --- | --- | --- | --- |
|  | **Male (N = 940)** | **Female (N = 519)** | **Male (N = 1,287)** | **Female (N = 1,292)** |
| **Age, years** | 60.3 ($\pm$11.3) | 63.0 ($\pm$11.2) | 64.3 ($\pm$16.6) | 63.7 ($\pm$15.0) |
| **Height, cm** | 168.6 ($\pm$6.1) | 158.2 ($\pm$6.4) | 167.1 ($\pm$11.7) | 155.2 ($\pm$9.9) |
| **Weight, kg** | 72.9 ($\pm$11.5) | 60.5 ($\pm$9.4) | 68.1 ($\pm$13.9) | 56.9 ($\pm$11.6) |
| **QRS-Duration, ms** | 93.9 ($\pm$10.8) | 86.3 ($\pm$9.6) | 90.3 ($\pm$11.8) | 82.7 ($\pm$10.6) |
| **P-Axis, degree** | 49.5 ($\pm$20.8) | 49.9 ($\pm$23.3) | 49.4 ($\pm$27.0) | 48.1 ($\pm$28.0) |
| **R-Axis, degree** | 28.5 ($\pm$35.4) | 36.8 ($\pm$34.2) | 33.2 ($\pm$45.3) | 38.6 ($\pm$40.9) |
| **T-Axis, degree** | 40.3 ($\pm$33.0) | 43.5 ($\pm$33.0) | 54.3 ($\pm$49.3) | 49.2 ($\pm$47.4) |

Supplementary Table 1. Characteristics for both datasets. Parameters are presented in the form of *mean (*$\pm$*standard deviation)*.

|  | **Performance metrics** | | | | | |
| --- | --- | --- | --- | --- | --- | --- |
| **Model name** | **Accuracy** | **Sensitivity** | **Specificity** | **PPV** | **F1 score** | **C-statistic** |
| **ECG-AI** | 0.68($\pm$0.03) | 0.35($\pm$0.07) | 0.80($\pm$0.01) | 0.40($\pm$0.04) | 0.37($\pm$0.04) | 0.70($\pm$0.02) |
| **LVM-AI** | 0.68($\pm$0.03) | 0.45($\pm$0.08) | 0.77($\pm$0.03) | 0.45($\pm$0.05) | 0.45($\pm$0.07) | 0.62($\pm$0.02) |
| **eLVMass-Net** | 0.77($\pm$0.03) | 0.47($\pm$0.04) | 0.89($\pm$0.03) | 0.65($\pm$0.06) | 0.54($\pm$0.04) | 0.70($\pm$0.02) |

Supplementary Table 2. Performance metrics of proposed prediction model and SOTA models in terms of LVH classification. LVH is defined as an indexed LVM >72 g/m^2^ for men or >55 g/m^2^ for women. All metrics are presented in the form of *mean (*$\pm$*standard deviation)*.

| **Model Name** | **MAE (g)** | **MAPE (%)** | **P-Value** |
| --- | --- | --- | --- |
| **ECG-AI** | 42.1 (±0.1) | 44.3 (±0.1) | *.03* |
| **LVM-AI** | 155.6 (±23.5) | 969.2 (±169.6) | *<.001* |
| **eLVMass-Net** | 29.2 (±0.6) | 20.3 (±0.7) | - |

Supplementary Table 3. External validation of the eLVMass-Net and the SOTA models on NTUH dataset (N = 2,579). P-values were computed to evaluate the statistical significance of MAPE differences between the proposed eLVMass-Net and two SOTA methods. Both MAE and MAPE are presented in the form of *mean (*$\pm$*standard deviation)*.

| **Model** | **Sex-Specific** |  | | |
| --- | --- | --- | --- | --- |
|  |  | **MAPE (%)** | **Relative Improvement (%)** | **P-Value** |
| **eLVMass-Net** | **None** | 12.9 ($\pm$1.1) | - | - |
|  | **Male** | 12.6 ($\pm$0.9) | 2.7 | *.30* |
|  | **Female** | 12.5 ($\pm$0.3) | 3.0 | *.10* |

Supplementary Table 4. The improvement and significance of sex-specific models. The relative improvement and the corresponding p-values were calculated by comparing the outcomes of the sex-specific models to those of the non-sex-specific models. MAPE values are presented in the form of *mean (*$\pm$*standard deviation)*.

|  | **Performance metrics** | | | | | |
| --- | --- | --- | --- | --- | --- | --- |
| **Model name** | **Accuracy** | **Sensitivity** | **Specificity** | **PPV** | **F1 score** | **C-statistic** |
| **eLVMass-Net**  **(non-sex-specific)** | 0.77($\pm$0.03) | 0.47($\pm$0.04) | 0.89($\pm$0.03) | 0.65($\pm$0.06) | 0.54($\pm$0.04) | 0.70($\pm$0.02) |
| **eLVMass-Net (male)** | 0.77($\pm$0.01) | 0.39($\pm$0.04) | 0.91($\pm$0.01) | 0.63($\pm$0.03) | 0.48($\pm$0.03) | **0.77(**$\pm$**0.05)^*^** |
| **eLVMass-Net (female)** | 0.75($\pm$0.05) | 0.46($\pm$0.15) | 0.90($\pm$0.04) | 0.69($\pm$0.05) | 0.54($\pm$0.13) | **0.75(**$\pm$**0.05)*** |

Supplementary Table 5. Performance metrics of non-sex-specific versus sex-specific eLVMass-Net in terms of LVH classification. LVH is defined as an indexed LVM >72 g/m^2^ for men or >55 g/m^2^ for women. All metrics are presented in the form of *mean (*$\pm$*standard deviation)*. *denotes *P<.01*.
